# Preferences Towards Electronically Exchanging Digital Images With Healthcare Providers Among US Adults

**DOI:** 10.7759/cureus.18770

**Published:** 2021-10-14

**Authors:** Grace Wei, Kea Turner, Kerry Hennessy, Lucia Seminario-Vidal

**Affiliations:** 1 Medicine, University of South Florida Morsani College of Medicine, Tampa, USA; 2 Health Outcomes and Behavior, Moffitt Cancer Center, Tampa, USA; 3 Dermatology, University of South Florida Morsani College of Medicine, Tampa, USA; 4 Cutaneous Oncology Program, Moffitt Cancer Center, Tampa, USA

**Keywords:** teledermatology, telemedicine, mhealth, digital, imaging

## Abstract

Background: The rapid expansion of telemedicine, including teledermatology, during the COVID-19 pandemic has required both providers and patients alike to adapt to this digital transition. However, patient attitudes towards electronically shared images with their providers are poorly understood. To address this gap, we assessed digital image sharing preferences and associated determinants in a nationally representative sample.

Methods: We analyzed pooled data from the Health Information National Trends Survey 4, Cycle 3 and 4. Digital image sharing preferences were compared by patient characteristics and beliefs via chi-square at a significance level of p<0.05, using sampling and jackknife replicate weights to develop nationally representative sample estimates and account for the complex survey design. P-values were adjusted for multiple comparisons when appropriate.

Results: Among 6437 adults, 53.5% reported reluctance in electronically shared images and videos with providers. Greater aversion was observed among adults aged 75 or above (70.9%), retired (67.3%), and those with lower education (65.1%), lower annual income (60.9%), limited English proficiency (63.3%), distrust in health information from doctors (75.4%), and fair or poor health (60.4%).

Conclusion: Patient hesitancy towards digital image sharing may present challenges for teledermatology adoption. Greater efforts may be needed to address the age and socioeconomic digital divide, multilingual telemedicine tools, and patient-physician dynamics to ensure vulnerable groups receive needed teledermatologic care.

## Introduction

The COVID-19 pandemic has catalyzed a rapid expansion of telemedicine, including teledermatology [1-5]. Due to its visual basis, dermatology, in particular, is primed for adapting to this digital transition. In addition, improvements in telehealth coverage, modalities, and out-of-state practice permissions have further increased the reach of teledermatology [3,4,6,7]. However, despite technological advancements in smart phones and mobile devices, patient apprehension remains a key barrier impacting telemedicine, even amidst the pandemic expansion [8]. The growth of teledermatology services will require patient buy-in and engagement, such as willingness to use personal devices to take and electronically share medical images with healthcare providers (HCPs). As such, an understanding of patient attitudes and determinants of these attitudes is necessary to address patient barriers to teledermatology adoption.

Prior research has generally been limited to on-site clinic studies, and patient attitudes towards exchanging digital images with HCPs via mobile devices from any location, including non-clinic settings, are poorly understood, and national estimates remain limited. Successful transitions towards teledermatology will benefit from an improved understanding of patient perspectives regarding digital image sharing. We sought to address this gap by evaluating digital image sharing preferences and assessing determinants of patient preferences among a nationally representative sample of US adults. This article was previously presented as a meeting abstract at the 2021 Society for Investigative Dermatology (SID) Annual Meeting on May 3^rd^ to May 8^th^, 2021.

## Materials and methods

We analyzed pooled data from the Health Information National Trends Survey (HINTS) 4, Cycle 3 and 4, a two-stage sampling cross-sectional survey of 6,437 (weighted: 452,034,848) civilian, non-institutionalized US adults administered by the National Cancer Institute between September to December 2013 and August to November 2014. To date, HINTS 4 Cycle 3 and 4 remain the most recent surveys to include a question on adult attitudes towards electronically exchanging images with providers and thus, assess digital image sharing preferences on a national scale. Patient characteristics, technology use, and health status and beliefs were correlated with responses to a survey question on how willing respondents were in exchanging digital images/videos (e.g., skin lesion photos) with HCPs electronically, including through mobile devices.

Our main outcome of interest was willingness or interest in electronically exchanging digital images or videos with a healthcare provider. Respondents of HINTS 4 Cycle 3 were asked, “How willing would you be to exchange the following types of medical information with a health care provider electronically through your mobile phone or tablet? Digital images/video (e.g., photos of skin lesions)” and in HINTS 4 Cycle 4 were asked, “How interested are you in exchanging the following types of medical information with a health care provider electronically? Digital images/video (e.g., photos of skin lesions).” Responses to these questions for both HINTS 4 Cycle 3 and 4 included “very,” “somewhat,” “a little”, and “not at all.” Demographics and health preferences, status, and beliefs of respondents are presented with frequencies and 95% confidence intervals. We compared group differences via chi-square at a significance level of p<0.05, reporting p-values adjusted for multiple comparisons when appropriate. Statistical analyses were conducted with SAS version 9.4 using sampling and jackknife replicate weights to develop nationally representative sample estimates and account for the complex survey design. Because this study involved de-identified publicly available data, approval from the University of South Florida Institutional Review Board was not sought or required.

## Results

Overall, 53.5% (95%CI, 51.1% to 56%) of US adults reported little to no willingness or interest towards digitally exchanging images or videos, such as skin lesions, with HCPs (Table 1). We observed significantly greater disinterest with increasing age, where 70.9% (95%CI, 66.4% to 75.4%, p<0.0001) of adults aged 75 or above stated little to no inclination to engage in this form of data sharing. We also found greater lack of interest among retired (67.3%, 95%CI, 64.2% to 70.5%, p<0.0001) and widowed adults (69.1%, 95%CI, 62.9% to 75.4%, p<0.0001). Notably, we observed a decreasing inclination towards digital image sharing with lower levels of education and income, with disinterest reported by 65.1% (95%CI, 57.8% to 72.4%, p<0.0001) of adults with less than a high school education and 60.9% (54.7% to 67%, p<0.0001) of adults with less than $20,000 annual income. In addition, adults who expressed speaking English not well or not at all were found to report reduced willingness or interest more frequently than adults who spoke English well or very well (63.3%, 95%CI, 54.9% to 71.7% versus 52.7%, 95%CI, 50.1% to 55.3%, p=0.031). Regionally, disinterest in digital image sharing was highest among states from the Midwest (57.1%, 95%CI, 53.0% to 61.1%, p=0.029). Adults located in counties with populations less than one million reported a greater lack of interest (57.3%, 95%CI, 53.4% to 61.2% versus 50.4%, 95%CI, 47.5% to 53.4%, p=0.018) compared to more largely populated areas (≥ 1 million). 

**Table 1:** Preferences for electronic exchange of digital images with HCPs, demographics *Indicate questions only present in HINTS 4 Cycle 3. **Indicate questions only present in HINTS 4 Cycle 4. Pooled sample size, n=6437, w=452,034,848 (n=sample size; w=weighted sample size). HCP: health care practitioner; adj: adjusted for multiple comparisons; USDA: United States Department of Agriculture.

Characteristics	Very, n=1508 w=114108000 % (95%CI)	Somewhat, n=1318 w=95960417 % (95%CI)	A little/Not at all, n=3611, w=241966431 % (95%CI)	P-Value	Adj
Total	25.2 (23.2-27.3)	21.2 (19.4-23.1)	53.5 (51.1-56)		
Age Group				< .0001>	< .0001>
18-34	31.5 (26.5-36.5)	22.3 (17.8-26.8)	46.2 (41.3-51.2)		
35-49	30.1 (26.8-33.4)	23.4 (19.9-26.8)	46.6 (42.3-50.8)		
50-64	20.4 (17.8-23.1)	20.5 (17.6-23.4)	59.1 (55.6-62.6)		
65-74	15.8 (13-18.7)	17.3 (14.5-20.1)	66.9 (64.1-69.7)		
75+	12.4 (9.5-15.3)	16.7 (13.1-20.3)	70.9 (66.4-75.4)		
Gender				0.039	0.041
Male	24.9 (21.5-28.4)	23.7 (20.8-26.6)	51.4 (47.6-55.1)		
Female	26.3 (23.6-29)	19.3 (17-21.5)	54.4 (51-57.7)		
Race/Ethnicity				0.091	0.240
Hispanic	29.2 (23.4-34.9)	21.1 (17.5-24.6)	49.8 (43.2-56.4)		
NH White	24.4 (21.8-26.9)	22.2 (19.7-24.7)	53.4 (50.6-56.3)		
NH Black/African-American	28.2 (22.6-33.7)	18.7 (13-24.4)	53.1 (45.9-60.3)		
NH American-Indian/Alaska native	22.8 (0-70.4)	3.2 (0-8.5)	74 (26.7-100)		
NH Asian	31.1 (19.7-42.5)	23.9 (14.8-33)	45 (34.8-55.2)		
NH native Hawaiian/Pacific islander	21.5 (0-64.2)	60.6 (0-100)	17.9 (0-61.5)		
NH multiple races	29.3 (12.8-45.9)	21.8 (8.5-35.1)	48.9 (31.4-66.3)		
Marital status				< .0001>	< .0001>
Married/Living as married	25.1 (23.2-27)	21.2 (19.3-23)	53.7 (51.3-56.2)		
Divorced/Separated	21.8 (17.4-26.3)	22.3 (17.4-27.1)	55.9 (50.5-61.3)		
Widowed	15.7 (11.2-20.2)	15.2 (8.9-21.5)	69.1 (62.9-75.4)		
Single, never been married	29.3 (23.5-35.1)	22.6 (17.3-27.8)	48.1 (42.4-53.8)		
Occupation Status				< .0001>	< .0001>
Employed	28 (25.6-30.3)	23.3 (21-25.6)	48.7 (45.8-51.7)		
Unemployed	27.7 (18.6-36.8)	14.8 (8.3-21.3)	57.5 (48.9-66)		
Homemaker	25.8 (20.4-31.3)	16 (11.5-20.4)	58.2 (51.1-65.3)		
Student	28.7 (14.2-43.2)	19.6 (8-31.2)	51.8 (37.9-65.6)		
Retired	14.8 (12.8-16.8)	17.8 (15.3-20.4)	67.3 (64.2-70.5)		
Disabled	23.1 (13.5-32.7)	17.5 (11-24)	59.5 (50-68.9)		
Other	33.8 (3.8-63.8)	16.5 (0-33.3)	49.7 (24.5-75)		
Education Status				< .0001>	< .0001>
Less than high school	18.6 (12.9-24.4)	16.3 (12.2-20.3)	65.1 (57.8-72.4)		
High school graduate	19.5 (16.1-22.8)	15.9 (12.5-19.3)	64.7 (60.5-68.8)		
Some college	24.5 (20.3-28.7)	23.3 (19.5-27.2)	52.2 (47.9-56.4)		
College graduate or more	31.3 (28.6-34.1)	23.7 (20.8-26.7)	44.9 (41.8-48)		
Income Status				< .0001>	< .0001>
Less than $20,000	22.1 (15.9-28.2)	17.1 (12.8-21.3)	60.9 (54.7-67)		
$20,000 to < $35,000	21.9 (16.4-27.5)	16.9 (11.3-22.6)	61.1 (54.5-67.8)		
$35,000 to < $50,000	25.8 (20.2-31.5)	19.3 (13.9-24.7)	54.9 (49.4-60.4)		
$50,000 to < $75,000	24.9 (20.2-29.6)	23.1 (18.4-27.9)	52 (46.8-57.1)		
$75,000 or more	31.6 (28.7-34.5)	25.9 (22.8-29.1)	42.5 (39-45.9)		
English Speaking				0.031	0.033
Not well or not at all	17.8 (11.1-24.5)	18.9 (12.7-25.1)	63.3 (54.9-71.7)		
Well or very well	25.9 (23.8-27.9)	21.5 (19.5-23.4)	52.7 (50.1-55.3)		
Census Region				0.016	0.029
Northeast	23.8 (20-27.6)	20.3 (15.7-24.9)	55.9 (50.7-61.1)		
Midwest	20.8 (17.5-24)	22.2 (18.7-25.6)	57.1 (53.0-61.1)		
South	26.6 (23.4-29.8)	20.1 (17-23.1)	53.3 (49-57.6)		
West	28.4 (24-32.7)	23 (19.8-26.1)	48.6 (44.2-53.1)		
USDA rural/urban designation (2003)				0.016	0.018
County Pop. ≥ 1 million	27.4 (24.9-29.8)	22.2 (19.7-24.7)	50.4 (47.5-53.4)		
County Pop. < 1 million	22.6 (19.4-25.8)	20.1 (17.5-22.6)	57.3 (53.4-61.2)		

We also noted significant differences in inclination towards digital image exchange based on health information preferences, including patterns of seeking health information and use and ownership of technology (Table 2). Adults who reported never seeking out health information (63.1%, 95%CI, 57.2% to 69%, p=0.002) or cancer information (53.7%, 95%CI 48.5% to 58.9%, p=0.004) also reported greater aversion to sharing digital images with HCPs. Further, 75.4% of adults (95% CI 65% to 85.7%, p=0.008) who reported little to no trust in health information received from a doctor also reported greater disinterest in digital image exchange. Never using the internet to seek out cancer information was associated with an increased prevalence of little to no interest in digital image exchange (69.2% 95%CI, 65% to 73.4% p<0.0001). We observed significantly greater disinterest in sharing digital images among adults who lacked mobile device ownership, including lack of owning a tablet (62.2%, 95%CI, 59.1% to 65.3%, p<0.0001) or smartphone (70.3%, 95%CI, 67.3% to 73.3%, p<0.0001), and highest among adults who did not own any devices (77.1%, 95%CI, 71.7% to 82.4%, p<0.0001). Even among device-users, those who lacked mobile health applications (48.3%, 95%CI, 44.1% to 52.5%, p=0.003) or who felt these applications did not contribute to their health decision-making (46.1%, 95%CI, 38.2% to 54%, p=0.031) reported greater reluctance towards digital image sharing.

**Table 2:** Preferences for electronic exchange of digital images with HCPs, technology use and health beliefs *Indicate questions only present in HINTS 4 Cycle 3. **Indicate questions only present in HINTS 4 Cycle 4. Pooled sample size, n=6437, w=452,034,848 (n=sample size; w=weighted sample size). HCP: health care practitioner; adj: adjusted for multiple comparisons; USDA: United States Department of Agriculture. No significant differences observed by insurance status and cancer history (p>0.05).

Characteristics	Very, n=1508 w=114108000 % (95%CI)	Somewhat, n=1318 w=95960417 % (95%CI)	A little/Not at all, n=3611, w=241966431 % (95%CI)	P-Value	Adj
Total	25.2 (23.2-27.3)	21.2 (19.4-23.1)	53.5 (51.1-56)		
Have sought health info previously	27.1 (24.7-29.4)	21.7 (19.7-23.8)	51.2 (48.4-54)	0.001	0.002
Trust Health Information From Doctors*				0.006	0.008
A lot	26.5 (21.7-31.4)	18.6 (15.7-21.5)	54.8 (50.5-59.1)		
Some	20.6 (15.9-25.2)	23.8 (15.6-32)	55.6 (47.9-63.4)		
A little or not at all	13.9 (4.6-23.2)	10.7 (4.5-16.9)	75.4 (65-85.7)		
Use internet for any reason	27.2 (24.9-29.5)	22.4 (20.2-24.6)	50.3 (47.7-53)	< .0001>	< .0001>
Device Ownership				< .0001>	< .0001>
Tablet	26.5 (18.9-34)	22.1 (14-30.3)	51.4 (43.8-59.1)		
Smartphone	27.1 (21.9-32.2)	24.4 (19.6-29.2)	48.5 (43.5-53.6)		
Basic cell phone only	11.3 (8.9-13.7)	13.8 (11.5-16.1)	74.9 (71.8-78)		
None of the above	11.7 (7-16.4)	11.2 (7.5-15)	77.1 (71.7-82.4)		
Multiple devices	32.2 (28.3-36)	24.5 (22-27)	43.4 (40.1-46.6)		
Have health apps on devices**	38.4 (31.6-45.2)	21.8 (17.9-25.8)	39.8 (33.5-46)	0.003	0.003
Health apps aided decision-making**	47.5 (35.4-59.6)	24.2 (15.9-32.4)	28.4 (19.2-37.5)	0.029	0.031
Medical Information Exchange Methods				< .0001>	< .0001>
E-mail only	42.4 (35.4-49.5)	23.4 (18.1-28.6)	34.2 (29.2-39.2)		
Text messaging only	32.1 (13.9-50.3)	22.7 (8.7-36.8)	45.2 (27.9-62.5)		
App(s) only	29.9 (5.3-54.5)	22.9 (6.8-39)	47.1 (26-68.3)		
Video conference only	15.9 (0-46.9)	30.3 (0-98.1)	53.8 (4.7-100)		
Social media only	13.1 (0-26.9)	32.9 (7.3-58.5)	54 (30.2-77.8)		
Fax only	22.4 (12.4-32.5)	24.9 (11-38.8)	52.7 (39-66.4)		
None of the above	19.7 (17.4-21.9)	20 (17.9-22.1)	60.3 (57.4-63.3)		
Multiple methods	40.7 (33.8-47.6)	25.2 (18-32.5)	34 (26.4-41.7)		
Confidence in Safeguards to Protect Medical Record Information				0.0003	0.0005
Very confident	31.1 (24.6-37.5)	15.5 (10.5-20.4)	53.5 (46.1-60.9)		
Somewhat confident	26.8 (23.3-30.3)	26.9 (23.5-30.3)	46.3 (42.4-50.2)		
Not confident	21.8 (16.1-27.5)	20.3 (15.1-25.4)	57.9 (52.5-63.3)		
Confidence That You Have Control in Who Can Use Your Medical Information				0.0005	0.0009
Very confident	31 (25.8-36.2)	17.1 (13.3-21)	51.9 (46.1-57.8)		
Somewhat confident	26.1 (21.8-30.3)	26.2 (23-29.5)	47.7 (44.1-51.3)		
Not confident	21.8 (17.4-26.2)	23.1 (17.3-29)	55 (48.9-61.1)		
Concern About Unauthorized Access in Sending Medical Information Electronically				< .0001>	< .0001>
Very concerned	16.8 (12.3-21.4)	18.9 (13.9-23.9)	64.3 (58.9-69.6)		
Somewhat concerned	26.7 (23.1-30.2)	25.3 (21.6-29)	48 (44.2-51.8)		
Not concerned	32.6 (27.4-37.9)	22.1 (17.3-26.9)	45.3 (40.1-50.4)		
General health				0.002	0.003
Very good or excellent	27.7 (24.2-31.2)	20.9 (18.4-23.5)	51.4 (48-54.8)		
Good	22.7 (19.8-25.6)	23.3 (20.8-25.8)	54 (50.3-57.7)		
Fair or poor	23.8 (18.9-28.8)	15.8 (12.8-18.8)	60.4 (55-65.7)		
Confidence in Ability to Take Good Care of Health				0.023	0.030
Very or completely confident	26.7 (24.1-29.3)	19.8 (17.8-21.9)	53.4 (50.6-56.3)		
Somewhat confident	22.4 (18.4-26.4)	25.6 (21.6-29.6)	52 (47.5-56.5)		
A little or not confident	20.7 (13.1-28.4)	15.8 (10.1-21.5)	63.4 (54.7-72.2)		
Family History of Cancer				0.004	0.006
Yes	25 (22.7-27.3)	22.9 (20.6-25.1)	52.2 (49.1-55.3)		
No	28 (23.7-32.2)	17.6 (15.1-20.1)	54.4 (50.6-58.2)		
Not sure	19.5 (13.8-25.2)	21 (12.2-29.8)	59.5 (51.3-67.6)		
Worry About Getting Cancer				< .0001>	0.0001
Not at all	21.7 (18.1-25.3)	16.4 (12.6-20.1)	62 (57.9-66.1)		
Slightly	24.1 (20.6-27.7)	25.1 (21.1-29.1)	50.7 (46.8-54.7)		
Somewhat	26.1 (21.7-30.4)	23.1 (20.2-26.1)	50.8 (46.2-55.4)		
Moderately	31.7 (25.3-38.2)	23.6 (17.1-30.1)	44.7 (38.1-51.3)		
Extremely	31.1 (24.4-37.9)	14.2 (9.4-19)	54.7 (47.1-62.2)		
Preferred Role in Treatment if Diagnosed With Cancer (Moderate Chance of Survival)**				0.021	0.048
My decision with little/no doctor input	20.3 (4-36.6)	10.1 (0-21.4)	69.6 (51.4-87.8)		
My decision with input from doctor	25.7 (20.3-31)	22.1 (18.3-25.8)	52.3 (47-57.5)		
Shared decision-making responsibility	25.8 (22.1-29.4)	25.9 (22.1-29.7)	48.4 (44.4-52.4)		
Doctors decision considering my opinion	32.1 (22.8-41.4)	18.8 (9.2-28.3)	49.1 (39.1-59.1)		
Leave treatment decisions to my doctor	29.5 (18-40.9)	11.6 (4.4-18.7)	59 (46.9-71)		
Preferred Role in Treatment if Diagnosed With Cancer (Low Chance of Survival)**				0.002	0.008
My decision with little/no doctor input	20.9 (11.6-30.2)	16.8 (6.6-27)	62.3 (50.6-74.1)		
My decision with Input from doctor	25.9 (21.2-30.6)	22.2 (18.5-26)	51.9 (47.2-56.5)		
Shared decision-making responsibility	26.2 (21.9-30.6)	27.2 (22.1-32.3)	46.6 (41.5-51.7)		
Doctors decision considering my opinion	31.8 (20.1-43.5)	12.1 (7.2-16.9)	56.2 (45.6-66.8)		
Leave treatment decisions to my doctor	29.1 (14.7-43.6)	15.6 (9.2-22)	55.3 (42.2-68.3)		
Frequency of Sunscreen Use				0.008	0.031
Always	27.7 (23.8-31.5)	18.6 (14.3-22.9)	53.7 (48.5-58.9)		
Often	27.6 (23.2-31.9)	22.3 (18-26.6)	50.1 (44.6-55.7)		
Sometimes	26.5 (22.3-30.7)	23.1 (19.6-26.6)	50.4 (46.7-54.2)		
Rarely	25.5 (20.1-30.9)	23.2 (18-28.3)	51.3 (44.8-57.8)		
Never	22.2 (17.9-26.5)	19.6 (15.8-23.4)	58.1 (53.7-62.6)		
Don't go out on sunny days	20.6 (8.1-33.1)	11.5 (5.2-17.8)	67.9 (52.3-83.4)		

Prior engagement in electronically exchanging medical information with HCPs and privacy concerns were associated with preferences towards digital image sharing. We observed a greater disinclination towards digital image sharing among adults who had not previously used electronic means to share health information with a provider (60.3%, 95%CI, 57.4% to 63.3%, p<0.0001). In addition, greater disinterest in digital image sharing was more prevalent among adults with privacy concerns regarding personal health data, with little to no willingness or interest reported by 57.9% (95%CI, 52.5% to 63.3%, p=0.0005) of adults who lack confidence in safeguards to protect their medical record information and 55% (95%CI, 48.9% to 61.1%, p=0.0009) of adults who lack confidence in personal control over who uses their medical information. Similarly, 64.3% (95%CI, 58.9% to 69.6%, p<0.0001) of adults who stated being very concerned about unauthorized access in electronic transmission of medical information were disinclined to share digital images with HCPs.

Finally, we observed significant differences in preference for digital image exchange with providers by health status and cancer beliefs. Aversion to digital image sharing was higher among adults describing their general health as only fair or poor (60.4%, 95%CI, 55% to 65.7%, p=0.003) and among adults with little to no confidence in personal ability to take good care of their health (63.4%, 95%CI, 54.7% to 72.2%, p=0.03). Disinterest in digital image sharing was also significantly more prevalent among adults who reported being not at all worried about getting cancer (62%, 95%CI, 57.9% to 66.1%, p<0.0001). Further, greater preferences for autonomous health decision-making were associated with reduced willingness or interest in digital image exchange. When asked about preferred roles in treatment if diagnosed with cancer with a moderate or low chance of survival, 69.6% (95%CI, 51.4% to 87.8%, p=0.048) and 62.3% (95%CI, 50.6% to 74.1%, p=0.008) of adults who reported preferring an autonomous decision with little to no input from their doctor also indicated disinclination towards imaging sharing with HCPs, respectively.

## Discussion

Our findings indicate that many US adults reported aversion to electronically exchanging digital images with HCPs, such as skin lesions, during 2013 and 2014. This contrasts with recent reports suggesting that nearly 50% of patients now opt for telemedicine over in-person visits, a 35% increase from pre-COVID-19 [1,2]. In addition to the clear impact of COVID-19, other contributing factors may also underly this discrepancy, including changing preferences over time, as well as sociodemographic factors and health beliefs. Indeed, we observed significant associations between disinclination towards digital image sharing and age, socioeconomic background, device ownership, language capabilities, privacy concerns, physician-patient relationship dynamics, poor health, lower self-care confidence, and reduced cancer risk-related distress. If the majority of current patients opting for telemedicine over in-person visits also align with factors that we have identified as favorable towards digital image sharing, such as younger age, higher socioeconomic status, and better health, this may further support a growing body of evidence revealing disparities in telemedicine [9,10]. This has significant implications for dermatologic care during the COVID-19 pandemic and post-COVID-19 era, as patient attitudes towards digital image sharing may represent a substantial barrier for seeking out and receiving teledermatology services. The COVID-19 pandemic has transitioned many dermatology practices towards providing the majority of care virtually, and even after clinics safely reopen, the integration of telehealth into dermatologic care will only continue to grow [11,12]. In addition, ongoing advances in guidelines and quality of teledermatology to a level comparable to in-person care further contribute to this expanding dermatologic digital shift [11, 13-15].

Our results are consistent with factors previously reported as contributing to a widening digital divide. Reluctance to engage in digital image sharing was significantly more prevalent among older age groups. This agrees with in-person clinic findings that older adults were more dissatisfied with sending medical images via personal smartphone to their provider [16] and with prior literature indicating that older adults are overall generally more hesitant to share personal health data [17,18], particularly via mobile devices [18]. Together, these results are undoubtedly impacted by the digital divide between older and younger age groups; increasing age is associated with significantly lower odds of possessing internet access and engaging in digital health activities [19-21]. Reluctancy among older populations towards sharing digital images is likely also associated with greater concerns regarding data misuse and security risks observed with increasing age [17]. Further, age-dependent digital competencies describe variations in mental models between older versus younger adults due to having grown up accustomed to different technologies [17]. 

Our results also indicated greater disinclination towards digital image sharing among groups from low socioeconomic backgrounds, with 60.9% of adults making less than $20,000 annually reporting disinterest in digital image sharing with HCPs. Similarly, we observed an increasing trend in disinterest with decreasing levels of education. Prior research demonstrates that adults with lower incomes and lower levels of education display a significantly reduced odds of using digital health services and owning mobile devices [22,23], and as such, may be less comfortable with using devices for mobile health (mHealth) purposes. Indeed, our findings demonstrate that non-owners of mobile devices report a significantly higher lack of interest or willingness to digitally share medical images with HCPs. Thus, these findings suggest that device ownership may not only impact patient ability to engage in mHealth-based teledermatology but likely also influences their attitudes toward engaging with these services as well. Improving access to telemedicine will likely be integral to mitigating reluctance towards digital image sharing with HCPs.

Study limitations include cross-sectional, self-reported data and limited generalizability as data was sampled during 2013 and 2014. Despite this, however, to our knowledge, HINTS 4 Cycle 3 and 4 remain the most recently available surveys to query digital image sharing preferences among a nationally representative sample of US adults; future studies in large datasets are needed to evaluate recent trends in digital image sharing preferences.

## Conclusions

Overall, many adults in the United States were reluctant to electronically exchange digital images or videos with providers in prior years. If these barriers persist, disinclination towards digital image sharing among certain groups will likely have significant implications for teledermatologic care during and after the COVID-19 pandemic and pose challenges to engaging with teledermatology services. Improved efforts targeting the age-impacted digital divide, mobile device access, interpreters and multilingual mHealth tools, secure mobile applications, and building trust with patients may be needed to address digital disparities and enhance the reach of teledermatology during this momentous expansion.
